# Seroprevalence of IgG Antibodies against *Echinococcus granulosus* by ELISA Method Using Recombinant Agb in Lorestan Province, Western Iran

**Published:** 2017-08

**Authors:** Vahid FALLAH OMRANI, Soheila ROUHANI, Bahram KAZEMI, Seyyed Javad SEYYEDTABAEI, Farnaz KHEIRANDISH, Maysam REZAPOUR

**Affiliations:** 1. Dept. of Parasitology and Mycology, School of Medicine, Shahid Beheshti University of Medical Sciences, Tehran, Iran; 2. Students Research Committee, Shahid Beheshti University of Medical Sciences, Tehran, Iran; 3. Dept. of Biotechnology, School of Advanced Technologies in Medicine, Shahid Beheshti University of Medical Sciences, Tehran, Iran; 4. Razi Herbal Medicines Research Center, Dept. of Parasitology and Mycology, School of Medicine, Lorestan University of Medical Sciences, Khorramabad, Iran; 5. Dept. of Epidemiology and Statistics, School of Public Health, Kerman University of Medical Sciences, Kerman, Iran

**Keywords:** Seroepidemiology, Cystic echinococcosis, Recombinant AgB, ELISA, Iran

## Abstract

**Background:**

Cystic echinococcosis (CE) is a zoonotic disease with global prevalence, which causes considerable health problems and economic losses throughout the world. The aim of this study was to assess the seroepidemiology of CE in Doroud City, Lorestan Province, Iran, considered a neglected endemic location.

**Methods:**

An ELISA was performed using recombinant AgB from Apr to Jul 2015 in Lorestan Province, Western Iran. The commercial Hydatidosis IgG ELISA kit (Vircell SL, Granada, Spain) was used to confirm the obtained results.

**Results:**

In the present study, out of 927 collected sera, 25 samples (2.6%) were found as seropositive for *E. granulosus* IgG antibodies. The prevalence of IgG antibodies against *E. granulosus* was significantly higher in rural areas (3.24%) than in urban area (1.20%) (*P*<0.001). Moreover, there was no significant relationship between age, occupation, sex, and literacy with seropositivity (*P*>0.05). Moreover, there was no statistically significant difference between the prevalence of CE in males (13/349, 3.72%) and females (12/553, 2.12%). With regard to occupation, farmers and ranchmen had the highest rate of infection (5.5%). There was a significant association between eating unwashed vegetables and seropositivity (*P*<0.001). Seropositive cases in rural areas were more than in urban areas.

**Conclusion:**

Since all the seropositive cases used unwashed local vegetables, the contamination may occur through the consumption of such vegetables.

## Introduction

Cystic echinococcosis (CE) is a cyclo-zoonotic disease caused by the larval form of the tape-worm *Echinococcus granulosus* (1). CE is prevalent worldwide, especially in regions of South America, North Africa, Europe, Siberia, China, Japan, and the Middle East (2).

*E. granulosus* is the cause of 95% of human CE worldwide. Echinococcosis occurs all over the world and causes economic losses and public health problems in many countries (1–4). In the endemic parts, the incidence rate of human CE has been recorded as >50 per 10^5^ person-years and as 5%–10% in parts of Peru, Argentina, East Africa, central Asia, and China (4, 5). Moreover, the incidence rate of CE has been recorded as 18–20 per 10^5^ in Turkey. In some countries such as parts of China, Central Asia, Eastern Europe and Israel, there is evidence and confirmed that the emergence or re-emergence of human CE has existed there for years (4). Moreover, in Europe, human CE occurs in every country or region, where the annual incidence rate of hospital cases varies between <1 and >8 per 10^5^ population; for example, in the northern part of Italy, the average annual CE incidence was recorded as 9.4-5.6/10^5^ inhabitants during 2003–2005 (6). In Iran, the incidence rate was reported to be 0.6–1.2 per 10^5^ population (7).

Definitive and intermediate hosts of *E*. granulosus are dogs and livestock, respectively. Human infection occurs due to the ingestion of contaminated food or water with infective parasite eggs or directly from infected canids. Epidemiologically, Iran is classified in two regions of hyperendemic (northern part) and endemic (southern part) in terms of CE (7). Human echinococcosis is approximately 1% of the admission rate to surgical wards in Iran (8, 9). Overall, 5%–45% of dogs are infected with *E.* granulosus in different areas in Iran (10). The overall direct and indirect annual costs of diagnosis, treatment, and control of CE are estimated to be the US $232.25 million in Iran, indicating the importance of the issue (10). Sixty percent of the infected rural population remains asymptomatic (7, 10).

Fresh vegetables are an important part of a healthy diet. Raw vegetables can be a transmission agent of intestinal parasites. Accordingly, many studies have reported contamination of raw vegetables with intestinal parasites (11–20).

Several studies have been conducted on the seroepidemiology of echinococcosis in different parts of Iran and other regions in the world (6, 21–26).

Despite several studies have already been performed on the frequency of different parasites (27–29) and the surgical cases of hydatid cyst in Lorestan Province, Iran (30), there are no data on the prevalence of CE in this province, especially in Doroud City. Results of this study might further clarify human CE in Iran.

## Materials and Methods

### Study area

**Fig. 1:**
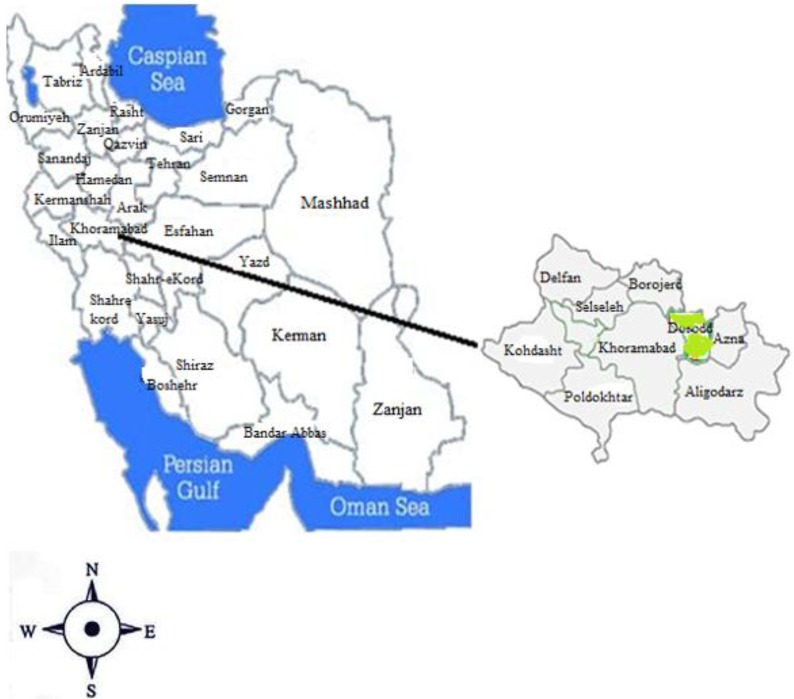
Studied location for sampling in Western Iran

This descriptive study was carried out from Apr to Jul 2015 in Doroud City, Lorestan Province, Iran. Doroud City is located in the east of Lorestan Province with an area of 1362 km^2^ (Fig. 1).

It has a mountainous temperate climate and average annual rainfall of 670 mm. According to the last census in 2013, the population of this city is 162800, of whom 100977 people are living in urban areas, and the rest in rural areas. One of the main occupations of people in the city is farming.

### Serum collection

Cluster sampling was used to collect the specimens. First, based on prevalence formula and *P*=0.1, d was calculated as 0.025 with 95% confidence interval for 600 samples. However, considering the design effect was increased about 1.5 times the number of samples, taking the loss, the final number increased to 927 samples. The samples were obtained from 31 clusters (almost 30 per cluster) related to 12 health care centers using the registry.

In the present study, 927 serum samples including 349 males and 553 females were collected. The serum samples were sent to parasitology and mycology laboratories. Completion of the questionnaires and collection of the samples were performed by individuals trained for this purpose. The obtained information was classified according to age group, sex, residence (rural/urban), occupation, education level, and unwashed raw local vegetable consumption.

### ELISA test

An ELISA was performed using recombinant AgB, as reported earlier (31). Briefly, microplates were coated with 2 μg/well of prepared recombinant AgB for 13 h at 4 °C. After three times of washing with phosphate-buffered saline (PBS)-Tween 20, the microplates were coated with a blocking solution (1% bovine serum albumin) for 90 min at room temperature. Then, the serum samples were coated, diluted (1:400) in PBS containing 0.1% Tween 20, and shaked for 2 h at room temperature. The microplates were again coated with anti-human IgG at the dilution of 1:10,000 for 1 h. O-phenylenediamine dihydrochloride (0.4 mg/ml) in 0.1 M phosphate/citrate buffer (pH5) and H_2_O_2_ was then added, incubated for 10 min, and stopped by the addition of 0.5 M H_2_SO_4_. The OD was registered at 490 nm (OD490) with an ELISA reader (Tecan, Switzerland). All the tests were performed in duplicate. According to the previous study, the cut-off was considered 0.47 (31). However, the commercial Hydatidosis IgG ELISA kit (Vircell SL, Granada, Spain) was used in accordance with the manufacturer’s protocol to confirm the results of recombinant antigen.

### Statistical analysis

The data were analyzed using the SPSS software (v. 16.5) (Chicago, IL, USA). Moreover, Fisher’s exact test was used for the qualitative part of the data analysis. To control the confounders, the logistic regression analysis was applied. Results were considered statistically significant when the *P*-value was less than 0.05.

## Results

Out of 927 collected sera, 25 (2.6%) samples were found as seropositive for *E. granulosus* IgG antibodies. The results of the commercial ELISA kit and recombinant antigen were similar. Descriptive characteristics are presented in Table 1. In the present study, the prevalence of IgG antibodies against *E. granulosus* was significantly higher in rural areas (3.24%) than in urban area (1.20%) (*P*<0.001). The maximum rate of CE was detected in the age group of <45 yr (2.7%) (Table 1). However, there was no significant relationship between CE seropositivity and age, occupation, sex, and education level. With regard to occupation, farmers and shepherds had the highest rate of seropositivity (5.5%), but no significant relationship was observed between CE seropositivity and occupation. Finally, in terms of unwashed raw local vegetable consumption, those who had consumed such vegetables demonstrated the significantly higher rate of seropositivity (3.97%) (*P*<0.001), as presented in Table 1.

**Table 1:** Seroepidemiology of hydatidosis according to epidemiological factors observed in Doroud city, Lorestan province, Western Iran, in 2015–2016

**Characteristic**	**Number**	**Seroprevalence% (95% CI)**	**Univariate analysis**		**Multivariate analysis**	
			**Odds ratio (95% CI)**	***P* –value**	**Odds ratio (95% CI)**	***P* s–value**
**Sex**			1.71(0.77-3.80)	0.18	1.91 (0.58–6.25)	0.28
Female^1^	565	12(2.12)				
Male	362	13(3.72)				
**Residence**			2.75 (0.82–9.27)	(P<0.001)	1.90(0.45–8.03)	0.38
Urban^1^	249	3(1.20)				
Rural	678	22(3.24)				
**Occupation**						
Farmer^1^	36	2(5.6)				
Student	344	8(2.3)	0.94 (0.8 – 11.13)	0.96	0.71 (0.10 – 50.04)	0.87
Official	19	1(5.3)	0.41 (0.083 –1.98)	0.26	0.79 (0.075 –8.35)	0.84
Others	528	14(2.7)	0.46 (0.1 - 2.12)	0.32	0.59(0.056 – 6.40)	0.66
**Literate**						
Illiterate^1^	235	5 (2.12)				
Primary	571	17 (2.97)	1.03(0.39–2.69)	0.95	0.48 (0.10 –2.30)	0.36
Diploma	95	2 (2.1)	1.24 (0.31 – 5.12)	0.76	1.17(0.18 – 7.63)	0.86
Upper diploma	26	1 (3. 84)	1.052 (0.17 –13.19)	0.70	0.32 (0.45 –8.03)	0.57
**Eating unwashed raw local vegetables**			228.3 (70.05 – 774.2)	0.000	313.3(79.37- 1237.4)	0.000
No^1^	908	11 (1.2)				
Yes	19	14 (73.7)				
**Age group**			0.89 (0.30 –2.63)	0.83	0.22 (0.03 –1.74)	0.15
<45^1^	764	21(2.7)				
≥45	163	4(2.5)				

## Discussion

In this study, recombinant AgB was applied for detecting seropositive patients. Sensitivity and specificity of this antigen were reported 91.66% and 97.22%, respectively (31).

In the present research, seroprevalence of IgG antibodies against *E. granulosus* was determined as 2.6% (25/927 cases), which is less than the rate reported in some similar studies conducted in Iran (24–26, 32, 33), but more than that in some other studies (34, 35).

One risk factor identified in this study was local vegetables. Multivariate analysis of data demonstrated that eating unwashed raw local vegetables and living in the rural areas are two risk factors for the infection.

Besides, a significant difference was observed between rural and urban life, as people in the rural areas revealed an increased rate of seropositive that is closely related to *E. granulosus* biological cycle. In India and China, greater seropositive prevalence has been reported in rural areas, as compared to urban areas (22, 34).

We found no significant relationship between sex and seropositivity, while some previous studies have suggested that females were more infected (5, 36–38).^.^ Similar seropositivity level among males and females in this study indicated that situations were likely similar in both the sexes.

Although seropositivity level was not significantly different in various age groups, the highest rate of seropositivity (2.7%) was observed in the age group <45-year-old, without any remarkable symptom.

In Iran, due to climate variations, prevalence and transmission of diseases occur differently. Lorestan Province, due to its favorable weather conditions, is a fertile ground for the spread of parasitic infections. Moreover, this province is one of the main areas for development and distribution of medicinal plants and it’s by-products. Due to its special geographic location, natural peaks and valleys, numerous surface waters, underground water tanks and more than 1.5 million hectares of forest and grassland, Lorestan province has extremely diverse vegetation. This province has 151 species of medicinal plants and thus, using local vegetables is common among the indigenous. In addition, one of the sources of income in rural areas is the cultivation and selling of plants.

However, lack of sanitation facilities while washing vegetables and close proximity of some of the vegetable fields to rivers are the concern. Based on the results of present research, a significant association was observed between local vegetable consumption and risk of hydatid cyst. Several studies have been conducted on vegetable contamination with parasites. In a study conducted in Tripoli, contamination of salad vegetables sold at wholesale and retail markets with some parasites was detected. Parasitological contamination of raw salad vegetables can be a source of human infection (11). In Shahrekord city, Iran, parasites were detected in unwashed vegetables (12). The results showed the potential of unwashed raw vegetables in the transmission of intestinal parasites to human, and also emphasized the use of proper washing and disinfecting procedures prior to consumption (12). In Burdur, Turkey, helminth eggs in raw vegetables were fund (14). The findings of this research demonstrated the importance of raw vegetables in the transmission of intestinal parasites and highlighted the role of raw vegetables in threatening public health (15).

In Iran, especially within the study area, there is an intention to eat raw vegetables during main meals. Due to the presence of multiple risk factors for infection in the study area, serious measures for prevention and control should be taken by health officials.

## Conclusion

Contaminated vegetables with parasite eggs may be one of the ways of infection transmission in this region. Raising awareness towards the adoption of different procedures to prevent transmission of the disease and reduce health care costs in the future appears to be necessary. Besides, due to the importance of this disease regarding health and economy, it is necessary to effectively and efficiently control the disease in Lorestan Province. Due to the development of animal husbandry in the region, implementing anti-parasitic treatments for sheepdogs, safe and sanitary disposal of infected organs, and consumption of washed vegetables are recommended to reduce the rate of infection.

## Ethical considerations

Ethical issues (Including plagiarism, informed consent, misconduct, data fabrication and/or falsification, double publication and/or submission, redundancy, etc.) have been completely observed by the authors.
